# Analysis of Gender Differences in Genetic Risk: Association of TNFAIP3 Polymorphism with Male Childhood-Onset Systemic Lupus Erythematosus in the Japanese Population

**DOI:** 10.1371/journal.pone.0072551

**Published:** 2013-08-30

**Authors:** Keisuke Kadota, Masaaki Mori, Masakatsu Yanagimachi, Takako Miyamae, Takuma Hara, Taichi Kanetaka, Tomo Nozawa, Masako Kikuchi, Ryoki Hara, Tomoyuki Imagawa, Tetsuji Kaneko, Shumpei Yokota

**Affiliations:** 1 Department of Pediatrics, Yokohama City University School of Medicine, Kanazawa-ku, Yokohama, Kanagawa, Japan; 2 Biobank, Department of Research Support and Coordination, Advanced Medical Research Center, Yokohama City University, Kanazawa-ku, Yokohama, Kanagawa, Japan; University of Chicago, United States of America

## Abstract

**Background:**

Systemic lupus erythematosus (SLE) is a systemic multisystem autoimmune disorder influenced by genetic background and environmental factors. Our aim here was to replicate findings of associations between 7 of the implicated single nucleotide polymorphisms (SNPs) in *IRF5*, *BLK*, *STAT4*, *TNFAIP3*, *SPP1*, *TNIP1* and *ETS1* genes with susceptibility to childhood-onset SLE in the Japanese population. In particular, we focused on gender differences in allelic frequencies.

**Methodology/Principal Findings:**

The 7 SNPs were genotyped using TaqMan assays in 75 patients with childhood-onset SLE and in 190 healthy controls. The relationship between the cumulative number of risk alleles and SLE manifestations was explored in childhood-onset SLE. Logistic regression was used to test the effect of each polymorphism on susceptibility to SLE, and Wilcoxon rank sum testing was used for comparison of total risk alleles. Data on rs7574865 in the *STAT4* gene and rs9138 in *SPP1* were replicated for associations with SLE when comparing cases and controls (corrected P values ranging from 0.0043 to 0.027). The rs2230926 allele of *TNFAIP3* was associated with susceptibility to SLE in males, but after Bonferroni correction there were no significant associations with any of the other four SNPs in *IRF5*, *BLK*, *TNIP1* and *ETS1* genes. The cumulative number of risk alleles was significantly increased in childhood-onset SLE relative to healthy controls (P = 0.0000041). Male SLE patients had a slightly but significantly higher frequency of the *TNFAIP3* (rs2230926G) risk allele than female patients (odds ratio [OR] = 4.05, 95% confidence interval [95%CI] = 1.46–11.2 P<0.05).

**Conclusions:**

Associations of polymorphisms in *STAT4* and *SPP1* with childhood-onset SLE were confirmed in a Japanese population. Although these are preliminary results for a limited number of cases, *TNFAIP3* rs2230926G may be an important predictor of disease onset in males. We also replicated findings that the cumulative number of risk alleles was significantly increased in childhood-onset SLE.

## Introduction

Systemic lupus erythematosus (SLE) is an autoimmune connective tissue disease which presents with various clinical symptoms and disorders in many internal organs. Disease onset in about 60% of patients occurs between the ages of 16 and 55 years, and approximately 15–20% are diagnosed in adolescence [1]. SLE occurs predominantly in women, with a female-to-male ratio of 10∶1. However, for childhood-onset SLE, this ratio is lower, at about 3∶1.

Childhood-onset SLE represents 10–20% of all SLE cases, and is associated with greater disease severity than adult-onset SLE, including more rapid renal damage. The majority of childhood-onset SLE patients will have developed renal damage within 5–10 years of disease onset [2]. Other studies of clinical characteristics indicated that men younger than 33 at the time of diagnosis were more likely to develop lupus nephritis, and that patients of Asian descent tend to have more severe disease, including the renal manifestations [3]. Recent large-scale association studies revealed a relationship between genetic risk factors and patient age at disease onset [4]. Childhood-onset SLE is associated with more frequent occurrence of the various SLE symptoms than seen in adult-onset disease, and has a more serious disease course [1]. Relative to adult-onset SLE, there is a higher genetic risk in childhood-onset SLE; this influences age of disease onset, which is an important predictor of disease severity [4]. Therefore, we hypothesize that gender-specific genetic effects of childhood-onset SLE are involved in its etiology. To test this hypothesis, here we explore the distribution of known susceptibility loci in a cohort of Japanese childhood-onset SLE patients.

Recent studies have identified the following genes as affecting susceptibility to SLE in Japanese: human leukocyte antigen DRB1 (*HLA-DRB1*) *15∶01, interferon regulatory factor 5 (*IRF5*) rs41298401C, signal transducer and activator of transcription-4 (*STAT4*) rs7574865T, B lymphoid tyrosine kinase (*BLK*) rs13277113A, tumor necrosis factor alpha-induced protein 3 (*TNFAIP3*) rs2230926G and TNFAIP3-interacting protein 1 (*TNIP1*) rs7708392C [5]–[10]. The same risk alleles are associated with SLE in the Caucasian and Chinese populations [11]–[14]. We previously evaluated associations of polymorphisms in *IRF5*, *BLK*, *STAT4*, *TNFAIP3* and *TNIP1* genes with susceptibility to SLE in Japanese children [7], [9].

Osteopontin (*SPP1*: secreted phosphoprotein 1) located on 4q22, also called early T lymphocyte activation 1, is a bone matrix mediator with important roles in inflammation and immunity [15]. An association between *SPP1* genetic polymorphisms and increased osteopontin protein levels has been reported [16]. Two *SPP1* single nucleotide polymorphisms (SNPs) (rs1126772 and rs9138) in the 3′ untranslated region were found to be associated with autoimmune/lymphoproliferative syndrome (ALPS). Osteopontin serum levels are associated both with these polymorphisms and an elevated risk of developing ALPS [17]. The same investigators reported that rs7687316 and rs9138 were significantly associated with SLE in an Italian population [18]. Another report implicated rs1126616 and rs9138 in conferring a significantly higher risk of SLE on men than women [19]. Therefore, it was suggested that rs9138 in the *SPP1* gene is a specific autosomal gene in male SLE.

E26 transformation-specific 1 (*ETS1*) is a member of the E26 transformation-specific family of transcription factors. *ETS1* plays a role in the negative regulation of Th17 cells and in B cell differentiation [20]. Recently, SLE susceptibility genes have been identified in two genome-wide association studies (GWAS) in Asian populations. It was found that genetic variants in *ETS1* were associated with susceptibility to SLE; reduced expression of *ETS1* may increase differentiation and activity of both plasma cells and Th17 cells [21], [22]. Two SNPs in *ETS1* (rs6590330 and rs4937333) were found to be significantly related to SLE, and reported to be associated with age of onset [11].

Recently, an association of the cumulative number of risk alleles and SLE has been reported in the United States and Japan [4], [23]. Koga et al. analyzed the cumulative number of risk alleles at eight established susceptibility loci, namely *HLA-DRB1*, *IRF5*, *STAT4*, *BLK*, *TNFAIP3*, *TNIP1*, *FCGR2B* and *TNFSF13*, in a Japanese population. Patients were found to carry more risk alleles than healthy controls. The odds ratio (OR) for SLE was significantly increased in individuals carrying 10 or more risk alleles. In contrast, subjects with 4 or less risk alleles had significantly decreased SLE occurrence.

The present study was conducted to determine whether the 7 candidate genes *IRF5*, *BLK*, *STAT4*, *TNFAIP3*, *SPP1*, *TNIP1* and *ETS1* were associated with childhood-onset SLE in the Japanese population. We investigated sex-specific differences in allelic frequencies and the cumulative number of risk alleles. We further examined the relationship between genetic risk for SLE and many clinical manifestations of the disease.

## Materials and Methods

### Ethics Statement

This study was performed in accordance with the Declaration of Helsinki and approved by the Ethics Committee of Yokohama City University School of Medicine. Written informed consent was given by parents/guardians for all children that participated in this study.

### Subjects

The aim of this study was to explore associations between SNPs in the seven genes *IRF5*, *BLK*, *STAT4*, *TNFAIP3*, *SPP1*, *TNIP1* and *ETS1* and susceptibility to childhood-onset SLE in a Japanese population. The distribution of SNPs in these genes confirmed that they acted as SLE susceptibility loci (Table S1) [6]–[11], [19]. We analyzed the genotype data at the seven risk SNPs and investigated associations between cumulative number of risk alleles and susceptibility to SLE. In addition, we examined gender differences in allelic frequencies and cumulative risk for SLE. Moreover, we investigated associations between these SNPs and the different clinical characteristics of the patients.

### Study Population and Clinical Data

A total of 75 children, 15 males (20.0%) and 60 females (80.0%), enrolled in this study were followed-up at Yokohama City University Hospital between December 2007 and December 2010. The mean age of patients was about 12 years at onset of SLE (Table 1). All patients fulfilled the American College of Rheumatology (ACR) Criteria score for SLE of 4 or higher [24], [25]. We explored gender differences in the clinical manifestations (malar rash, arthritis, serositis, thrombocytopenia, proteinuria and anti-dsDNA antibodies, according to the ACR classification criteria).

**Table 1:** Demographic characteristics of the SLE population studied.

Age		yrs.
	Range	3–18
	Mean	11.9
**Gender**		**n (%)**
	Males	15 (20.0)
	Females	60 (80.0)

### Genotyping

SNPs in the *BLK* (rs13277113), *STAT4* (rs7574865), *TNFAIP3* (rs2230926), *SPP1* (rs9138), *TNIP1* (rs7708392) and *ETS1* (rs4937333) genes were selected based on previous research [7]–[10], [21], [26]. Seventy five patients with SLE and 190 healthy controls were genotyped. Genomic DNA was isolated from peripheral blood using the QIAamp DNA Mini kit (Qiagen K.K, Tokyo, Japan). Genotyping was performed using TaqMan SNP Genotyping Assays (Applied Biosystems, Foster, CA, USA assay ID: C___1886916_10 for rs13277113, C__29882391_10 for rs7574865, C___7701116_10 for rs2230926, C___8826997_10 for rs9138, C__29349759_10 for rs7708392, C____278298_10 for rs4937333). We developed a custom-made assay for rs41298401 in *IRF5* (forward primer, 5′-GCTGCGCCTGGAAAGC-3′; reverse primer, 5′-GGGAGGCGCTTTGGAAGT-3′; probes, VIC-CTGCTGTAGGCACCC and FAM-CTGCTGTAGCCACCC; Custom TaqMan Applied Biosystems, Foster, CA, USA). To confirm the accuracy of genotyping, we conducted bidirectional sequencing on 6 randomly selected individuals for each genotype at rs41298401 and undertook direct sequencing of the SNP. No genotyping errors were found. These SNPs were analyzed by real-time PCR using the AB7500 Real Time PCR system (Applied Biosystems, Foster, CA, USA) under the conditions recommended by the manufacturer.

### Statistical Analysis

Hardy-Weinberg Equilibrium (HWE) was measured by chi-squared testing for the control groups of each study (significance set at P<0.05). All analyses were performed using IBM SPSS software (SPSS for Windows, Version 19.0, Chicago, IL, USA). The multiple comparison P-values were corrected by the Bonferroni method, in which P values were multiplied by the number of tested SNPs, and P<0.05 considered significant (2-sided test).

We compared the clinical manifestations of SLE cases between male and female patients using Fisher’s exact probability test. Logistic regression was used to test the effect of each of the 7 SNPs on differences between SLE cases and healthy controls. A genetic additive model (with values: AA = 2, Aa = 1 and aa = 0, respectively) was considered. We analyzed the correlation between the risk for each SNP using a dominant or recessive model in logistic regression analysis. Dominant (risk allele homozygotes = 1, heterozygotes = 1 and nonrisk allele homozygotes = 0) and recessive (risk allele homozygotes = 1, heterozygotes = 0 and nonrisk allele homozygotes = 0) models were tested. Results are expressed as odds ratios (OR) with 95% confidence intervals (CI). Comparison of male and female patients used logistic regression [27], which was also used to test the effect of each polymorphism separately. Gender-specific associations were assessed by comparing the additive model between cases and controls for each gender separately.

The cumulative number of risk alleles was determined in each patient. Wilcoxon rank sum testing was used for comparisons of groups.

## Results

### Study Population and Clinical Data

We studied 75 cases and 190 controls. The median age at SLE diagnosis was 11.9 years (Table 1). There were 15 male and 60 female patients. All patients and controls (male n = 91, female n = 99) were Japanese. Female gender was a risk factor for SLE susceptibility (P<0.001, Fisher’s exact test). However, proteinuria was higher in males than females with SLE (P = 0.026, Fisher’s exact test) (Table 2), while malar rash, arthritis, serositis, thrombocytopenia and the presence of anti-dsDNA antibodies were not significantly difference between the sexes.

**Table 2:** The clinical and serological manifestations of patients with SLE.

		All	Male (n = 15)	Female (n = 60)	*P* value*
		N (% of group)	N (% of group)	N (% of group)	
Malar rash	+	49 (76.6)	11 (91.7)	38 (73.1)	0.27
	−	15 (23.4)	1 (8.3)	14 (26.9)	
Arthritis	+	39 (60.0)	6 (50.0)	33 (62.3)	0.52
	−	26 (40.0)	6 (50.0)	20 (37.7)	
Serositis	+	4 (6.3)	1 (10.0)	3 (5.7)	0.51
	−	59 (93.7)	9 (90.0)	50 (94.3)	
Thrombocytopenia	+	19 (27.5)	3 (23.1)	16 (28.6)	1.00
	−	50 (72.5)	10 (76.9)	40 (71.4)	
Proteinuria	+	47 (65.3)	13 (92.9)	34 (58.6)	**0.026**
	−	25 (34.7)	1 (7.1)	24 (41.4)	
Anti-dsDNA	+	55 (93.2)	11 (91.7)	44 (93.6)	1.00
	−	4 (6.8)	1 (8.3)	3 (6.4)	

* Fisher’s exact p value.

### Associations between the 7 Tested SNPs and Susceptibility to SLE

We conducted an analysis of Hardy-Weinberg equilibrium and genotypic and allelic associations of 7 SNPs with susceptibility to SLE. The genotype frequencies for the SNPs in both patients with SLE and healthy controls were in Hardy-Weinberg equilibrium (P>0.05).

A significant difference in allele distribution of *STAT4* rs7574865T between SLE patients and controls (OR = 1.77, 95%CT = 1.20–2.62, P = 0.027) was observed (Table 3). The genotype frequency of rs9138 in *SPP1* was also significantly different between SLE patients and controls (OR = 3.31, 95%CI = 1.67–6.56, P = 0.0043) in the recessive model (AA vs AC/CC) (Table 3). The genotype AA was significantly higher in patients than in controls.

**Table 3:** Associations of the seven SNPs with SLE in a Japanese population.

						Allele association		Dominant model		Recessive model	
	SNPs	Genotype			Allele	OR (95% CI)	*P* *	OR (95% CI)	*P* *	OR (95% CI)	*P* *
***IRF5***	**rs41298401C**	**CC**	**CG**	**GG**	**C**						
	Case (n = 75)	56 (74.7)	18 (24.0)	1 (1.3)	130 (86.7)	1.21 (0.73–2.02)	n.s.	4.11 (0.52–32.7)	n.s.	1.08 (0.59–1.99)	n.s.
	Control (n = 190)	139 (73.2)	41 (21.6)	10 (5.3)	319 (83.9)						
***BLK***	**rs13277113A**	**AA**	**AG**	**GG**	**A**						
	Case (n = 75)	43 (57.3)	28 (37.3)	4 (5.3)	114 (76.0)	1.54 (0.98–2.42)	0.41	1.63 (0.53–5.05)	n.s.	1.70 (0.99–2.91)	0.39
	Control (n = 190)	84 (44.2)	90 (47.4)	16 (8.4)	258 (67.9)						
***STAT4***	**rs7574865T**	**TT**	**TG**	**GG**	**T**						
	Case (n = 75)	16 (21.3)	39 (52.0)	20 (26.7)	71 (47.3)	1.77 (1.20–2.62)	**0.027**	2.27 (1.27–4.09)	0.042	1.97 (0.97–3.98)	0.41
	Control (n = 190)	23 (12.1)	81 (42.6)	86 (45.3)	127 (33.4)						
***TNFAIP3***	**rs2230926G**	**GG**	**GT**	**TT**	**G**						
	Case (n = 75)	3 (4.0)	12 (16.0)	60 (80.0)	18 (12.0)	2.02 (1.07–3.81)	0.21	1.82 (0.89–3.71)	0.70	INF**	
	Control (n = 190)	0 (0.0)	23 (12.1)	167 (87.9)	23 (6.1)						
***SPP1***	**rs9138A**	**AA**	**AC**	**CC**	**A**						
	Case (n = 75)	21 (28.0)	29 (38.7)	25 (33.3)	71 (47.3)	1.61 (1.10–2.38)	0.11	1.28 (0.73–2.24)	n.s.	3.31 (1.67–6.56)	**0.0043**
	Control (n = 190)	20 (10.5)	96 (50.5)	74 (38.9)	136 (35.8)						
***TNIP1***	**rs7708392C**	**CC**	**CG**	**GG**	**C**						
	Case (n = 75)	40 (53.3)	35 (46.7)	0 (0.0)	115 (76.7)	1.06 (0.66–1.70)	n.s.	INF**		0.89 (0.52–1.52)	n.s.
	Control (n = 190)	107 (56.3)	74 (38.9)	9 (4.7)	288 (75.8)						
***ETS1***	**rs4937333T**	**TT**	**TC**	**CC**	**T**						
	Case (n = 75)	24 (32.0)	40 (53.3)	11 (14.7)	88 (58.7)	1.40 (0.95–2.04)	n.s.	2.08 (1.02–4.25)	0.32	1.28 (0.72–2.30)	n.s.
	Control (n = 190)	51 (26.8)	89 (46.8)	50 (26.3)	191 (50.3)						

* P values calculated by logistic regression analysis and then corrected by the Bongerroni criterion.

** infinity.

Genotype and allele frequencies are shown in parentheses (%).

n.s., not significant.

### Clinical Subsets

Next, we explored whether these risk alleles were associated with the development of particular clinical manifestations. Frequencies of the rs9138 A-allele in *SPP1* (OR = 0.48, 95%CI = 0.25–0.92, P = 0.028) and the rs4937333 T-allele in *ETS1* (OR = 2.28, 95%CI = 1.03–5.04, P = 0.042) were significantly increased in SLE patients with proteinuria as compared to those without. There were no significant associations between *TNFAIP3* rs2230926G and proteinuria in SLE patients. Malar rash was associated with *STAT4* rs7574865T (OR = 3.08, 95%CI = 1.21–7.85, P = 0.019), whereas no significant associations were observed in the other subsets of patients with arthritis, serositis, thrombocytopenia and anti-dsDNA antibodies.

### The Cumulative Number of Risk Alleles at the 7 SNPs and Gender Differences

We had selected these 7 established SLE susceptibility alleles due to their reported significance in Asian populations or because they conferred a higher risk of SLE to males than females (Table S1). The cumulative risk was calculated in each patient by summing the risk scores. It was found that the cumulative number of the risk alleles was significantly increased in patients with childhood-onset SLE relative to the healthy controls (P = 0.0000041, Wilcoxon rank sum test). In contrast, there were no significant associations with gender itself (P = 0.085, Wilcoxon rank sum test).

Secondly, we evaluated the effect of these SNPs separately on the gender difference in SLE susceptibility. A significant gender-gene interaction was observed for rs2230926, a polymorphism located in *TNFAIP3*. The frequency of the risk allele in *TNFAIP3* (rs2230926G) was slightly but significantly higher in men than women with SLE (OR = 4.05, 95%CI = 1.46–11.23, P<0.05) (Table 4), whereas there were no differences between men (n = 91) and women (n = 99) in the controls. To further assess the effect of *TNFAIP3* (rs2230926), we calculated associations with SLE in gender-specific case-control analysis. Rs2230926 was associated with SLE in males (case n = 15, control n-91) but not in females (case n = 60, control n = 99) (OR male = 6.17, 95%CI = 2.10–18.09, P = 0.0064 corrected; OR female = 1.25, 95%CI = 0.52–3.02, P = 0.62 uncorrected) (Table 5).

**Table 4:** Comparison between male and female patients with SLE.

						Allele association		
SNPs		Genotype			Allele	OR (95% CI)	*P* value	Corrected *P* value*
***IRF5***	**rs41298401C**	**CC**	**CG**	**GG**				
	Male (n = 15)	9 (60.0)	6 (40.0)	0 (0.0)	24 (80.0)	0.51 (0.17–1.53)	0.23	n.s.
	Female (n = 60)	47 (78.3)	12 (20.0)	1 (1.7)	106 (88.3)			
***BLK***	**rs13277113A**	**AA**	**AG**	**GG**				
	Male (n = 15)	8 (53.3)	6 (40.0)	1 (6.7)	22 (73.3)	0.83 (0.33–2.10)	0.70	n.s.
	Female (n = 60)	35 (58.3)	22 (36.7)	3 (5.0)	92 (76.7)			
***STAT4***	**rs7574865T**	**TT**	**TG**	**GG**				
	Male (n = 15)	5 (33.3)	9 (60.0)	1 (6.7)	19 (63.3)	2.40 (1.00–5.81)	0.051	0.36
	Female (n = 60)	11 (18.3)	30 (50.0)	19 (31.7)	52 (43.3)			
***TNFAIP3***	**rs2230926G**	**GG**	**GT**	**TT**				
	Male (n = 15)	2 (13.3)	5 (33.3)	8 (53.3)	9 (30.0)	4.05 (1.46–11.23)	0.0071	**<0.05**
	Female (n = 60)	1 (1.7)	7 (11.7)	52 (86.7)	9 (75.0)			
***SPP1***	**rs9138A**	**AA**	**AC**	**CC**				
	Male (n = 15)	2 (13.3)	7 (46.7)	6 (40.0)	11 (36.7)	0.64 (0.30–1.36)	0.24	n.s.
	Female (n = 60)	19 (31.7)	22 (36.7)	19 (31.7)	60 (50.0)			
***TNIP1***	**rs7708392C**	**CC**	**CG**	**GG**				
	Male (n = 15)	10 (66.7)	5 (33.3)	0 (0.0)	25 (83.3)	2.00 (0.61–6.55)	0.25	n.s.
	Female (n = 60)	30 (50.0)	30 (50.0)	0 (0.0)	90 (75.0)			
***ETS1***	**rs4937333T**	**TT**	**TC**	**CC**				
	Male (n = 15)	7 (46.7)	7 (46.7)	1 (6.7)	21 (70.0)	2.00 (0.79–5.00)	0.14	n.s.
	Female (n = 60)	17 (28.3)	33 (55.0)	10 (16.7)	67 (55.8)			

* P values calculated by logistic regression analysis and then corrected by the Bongerroni criterion.

Genotype and allele frequencies are shown in parentheses (%).

n.s., not significant.

**Table 5:** Associations of SNPs with SLE in male and female groups separately assessed by logistic regression.

			Genotype						Allele		Allele association	
SNPs			Case (male n = 15, female n = 60)			Control (male n = 91, female n = 99)			Case	Control	OR (95% CI)	* *P* value
***IRF5***	**rs41298401C**		**CC**	**CG**	**GG**	**CC**	**CG**	**GG**				
	Male		9 (60.0)	6 (40.0)	0 (0.0)	67 (73.6)	15 (16.5)	9 (9.9)	24 (80.0)	149 (81.9)	0.91 (0.40–2.11)	n.s.
	Female		47 (78.3)	12 (20.0)	1 (1.7)	72 (72.7)	26 (26.3)	1 (1.0)	106 (88.3)	170 (85.9)	1.26 (0.63–2.54)	n.s.
***BLK***	**rs13277113A**		**AA**	**AG**	**GG**	**AA**	**AG**	**GG**				
	Male		8 (53.3)	6 (40.0)	1 (6.7)	39 (42.9)	43 (47.3)	9 (9.9)	22 (73.3)	121 (66.5)	1.41 (0.58–3.44)	n.s.
	Female		35 (58.3)	22 (36.7)	3 (5.0)	45 (45.5)	47 (47.5)	7 (7.1)	92 (76.7)	137 (69.2)	1.51 (0.88–2.61)	n.s.
***STAT4***	**rs7574865T**		**TT**	**TG**	**GG**	**TT**	**TG**	**GG**				
	Male		5 (33.3)	9 (60.0)	1 (6.7)	13 (14.3)	40 (44.0)	38 (41.8)	19 (63.3)	66 (36.3)	3.03 (1.32–6.97)	0.062
	Female		11 (18.3)	30 (50.0)	19 (31.7)	10 (10.1)	41 (41.4)	48 (48.5)	52 (43.3)	61 (30.8)	1.71 (1.06–2.75)	0.19
***TNFAIP3***	**rs2230926G**		**GG**	**GT**	**TT**	**GG**	**GT**	**TT**				
	Male		2 (13.3)	5 (33.3)	8 (53.3)	0 (0.0)	11 (12.1)	80 (87.9)	9 (30.0)	11 (60.4)	6.17 (2.10–18.09)	**0.0064**
	Female		1 (1.7)	7 (11.7)	52 (86.7)	0 (0.0)	12 (12.1)	87 (87.9)	9 (75.0)	12 (60.6)	1.25 (0.52–3.02)	n.s.
***SPP1***	**rs9138A**		**AA**	**AC**	**CC**	**AA**	**AC**	**CC**				
	Male		2 (13.3)	7 (46.7)	6 (40.0)	8 (8.8)	46 (50.5)	37 (40.7)	11 (36.7)	62 (34.1)	0.88 (0.38–2.06)	n.s.
	Female		19 (31.7)	22 (36.7)	19 (31.7)	12 (12.1)	50 (50.5)	37 (37.4)	60 (50.0)	74 (37.4)	0.61 (0.39–0.97)	0.24
***TNIP1***	**rs7708392C**		**CC**	**CG**	**GG**	**CC**	**CG**	**GG**				
	Male		10 (66.7)	5 (33.3)	0 (0.0)	49 (53.8)	40 (44.0)	2 (2.2)	25 (83.3)	138 (75.8)	1.76 (0.58–5.31)	n.s.
	Female		30 (50.0)	30 (50.0)	0 (0.0)	58 (58.6)	34 (34.3)	7 (7.1)	90 (75.0)	150 (75.8)	0.96 (0.55–1.66)	n.s.
***ETS1***	**rs4937333T**		**TT**	**TC**	**CC**	**TT**	**TC**	**CC**				
	Male		7 (46.7)	7 (46.7)	1 (6.7)	26 (28.6)	45 (49.5)	20 (22.0)	21 (70.0)	97 (53.3)	2.06 (0.88–4.79)	0.67
	Female		17 (28.3)	33 (55.0)	10 (16.7)	25 (25.3)	44 (44.4)	30 (30.3)	67 (55.8)	94 (47.5)	1.39 (0.88–2.18)	n.s.

* P values calculated by logistic regression analysis and then corrected by the Bongerroni criterion.

Genotype and allele frequencies are shown in parentheses (%).

n.s., not significant.

## Discussion

SLE is a systemic multisystem autoimmune disorder, susceptibility to which is influenced by a complex genetic background and potentially by environmental exposures. Because chronologically, childhood has less time to accumulate environmental exposures, the impact of genetic factors may be easier to disentangle than in adult-onset SLE. Recent studies identified many susceptibility genes relevant to SLE. We have replicated results for 3 of these (*STAT4*, *TNFAIP3* and *SPP1*) in the present study, but we were unable to replicate the findings of susceptibility for four others (*IRF5*, *BLK*, *ETS1* and *TNIP1*). The frequencies of the risk alleles *IRF5*, *BLK*, *ETS1* and *TNIP1* in the present study are similar to those published in other recent Asian studies [6], [8], [10], [11]. We may not have seen a significant difference because of the small size of our study.

The cumulative number of risk alleles is reported to be significantly increased in SLE relative to healthy controls in the Japanese population [23]. We did replicate this association in Japanese childhood-onset SLE. A recent study of cumulative genetic risk scores for SLE in men and women concluded that there is a higher genetic risk factor in men [28] and that male patients had a higher frequency of clinical manifestations than women [29]. In the present study, we found no significant differences in the number of SLE risk alleles in male and female patients. Female predominance in SLE is clearly evident in general, but the male: female ratio is lower in children with prepubertal onset and onset recently after puberty than at reproductive ages [30]. We suggest that genetic susceptibility is more clearly associated with childhood-onset SLE than adult-onset SLE. We did not find a significant difference in the cumulative number of risk alleles between male and female SLE patients. Therefore, the increased frequency of adult-onset SLE among women may be attributed to differences in environmental factors and the metabolism of sex hormones in particular.

Our findings suggest that susceptibility risks conferred by polymorphisms in the three genes *STAT4*, *TNFAIP3*, *SPP1* participate in the pathogenesis of childhood-onset SLE, but identified the *TNFAIP3* rs2230926G allele as the only one significantly different in male and female patients. This finding suggests that *TNFAIP3* rs2230926G may be an important predictor of disease in males. However, this should still be considered a tentative conclusion because there were only 15 male patients in our study, and previous large-scale studies of *TNFAIP3* have not identified a similar gender effect. Therefore, a multicenter study of the same ethnic group is needed to validate the results from this study. A20 (also known as TNFAIP3) encodes a ubiquitin-editing protein that acts in the negative regulation of nuclear factor-kappaB (NF-κB) signaling via several pathways including those involving tumor necrosis factor (TNF) and Toll-like receptors (TLR) [31]. Resent genome-wide association studies (GWAS) have revealed associations between *TNFAIP3* and SLE [32]. The SNPs rs2230926 in *TNFAIP3* introduces the amino acid substitution Phe127 to Cys127. Previous studies demonstrated that *TNFAIP3* risk allele rs2230926G leads to reduced NF-κB inhibition. Levels of *TNFAIP3* mRNA are lower in carriers of the rs2230926 G-allele (GT subjects) than in non-carriers (TT), resulting in less protein expression and less A20 inhibitory activity [9], [33]. Further research is needed to determine the influence of gene polymorphisms in the *TNFAIP3* gene on the potential role of A20 protein in the pathogenesis of SLE.

A recent review reported that a high a percentage of childhood-onset SLE patients have renal involvement [34]. In the present study, we observed that *SPP1* rs9138C was associated with proteinuria in SLE patients (OR = 2.10, 95%CI = 1.09–4.08, P = 0.028). On the other hand, the frequency of *SPP1* rs9138C tended to be decreased in the SLE patients studied here (OR = 0.62, 95%CI = 0.42–0.91, P = 0.15). Comparing the data from our Japanese population with those previously published from a Caucasian population, the frequency of the C allele at rs9138 was found to be significantly increased [26]. *SPP1* rs9138C was reported to contribute to SLE susceptibility in a population including both African-American and European-American males, but not females [19]. It can be suggested that *SPP1* rs9138C is only associated with increased risk of renal disorders in childhood- but not adult-onset SLE patients. Kariuki et al reported age- and gender- dependent effects of *SPP1* (rs9138C) on cytokine profiles in SLE patients. The rs9138C allele is associated with higher serum osteopontin and interferon-alpha in male and female patients ≤23 years old. [35] These results are consistent with our findings.

Our study is the first to report an association between risk alleles and clinical manifestations in Japanese children with SLE. In particular, both geography and race affect the prevalence of SLE and the frequency and severity of clinical manifestations. Although our study is of a small single-center cohort, this minimizes any discrepancies in management and assessment, and it reflects the homogeneity of the ethnic and socio-demographic background. In addition, because it focuses exclusively on patients with childhood-onset SLE, it is more appropriate for defining detailed genetic susceptibility in this group than cohorts investigating all SLE patients. Our hospital is a pediatric rheumatology center treating many patients with severe SLE. However, we acknowledge that the current study suffers from some shortcomings. The sample size for male SLE patients is small (15 males). In addition, further research is needed to study relationships between adult-onset SLE and childhood-onset patients. Therefore, it is important that the association of *TNFAIP3* and *SPP1* polymorphisms with childhood-onset SLE susceptibility is confirmed by other groups.

## Conclusions

Associations of *STAT4* and *SPP1* with childhood-onset SLE were confirmed in a Japanese population. For *TNFAIP3*, this genetic effect may be confined to males. We replicated previous findings that the cumulative number of risk alleles of seven known SNPs was significantly increased in childhood-onset SLE relative to controls, while there was no association with the observed gender difference.

## Supporting Information

Table S1 **Single nucleotide polymorphisms (SNPs) representing the 7 systemic lupus erythematosus (SLE) susceptibility loci genotyped in our study.**
(XLS)
